# Review of "*Evolution in Health and Disease (2^nd ^Edition*)" by Stearns S.C. & Koella J.C. (Eds)

**DOI:** 10.1186/1756-3305-2-4

**Published:** 2009-01-07

**Authors:** Jerzy M Behnke

**Affiliations:** 1 School of Biology, University of Nottingham, University Park, Nottingham NG7 2RD, UK

## Review

One of the signs I always look for when reviewing a book based on multiple authors is for some element of uniformity in the structure of the contributed chapters. This is always a good indication that the Editors have done their job well; that is, they have taken an active interest in each of the contributions and helped to mould them into a common format. I cannot fault this book on these grounds: each chapter is very well organised. Indeed, most acknowledge and express thanks to the Editors for their suggestions. I liked the focused introductions which set out what was to follow and the concise, objective, numbered summary points with which each chapter ends.

Another hallmark of well written multi-author editions is the absence of repetition. This is indeed generally very difficult to avoid when each contributor wishes to introduce his/her theme as clearly and comprehensively as possible, with appropriate background. Well, there is some repetition (e.g. lactose intolerance was explained and discussed several times in the first set of chapters, as was the aging theme in the final set), but it is not irritating. Again, I suspect that this is because of the fine work by the Editors.

This book starts with a stunning chapter that grabs the reader's attention and sets out a direction for what then follows. I really enjoyed reading Chapter One entitled "Introducing evolutionary thinking for medicine". It is succinct, clear and illustrated with some beautiful examples that drive the points home. It is a good balance of readable text and relevant fact. This is an excellent introduction to the book and its contents, and it certainly made me eager to read on.

*Evolution in Health and Disease *(2^nd ^Edition) contains 23 chapters, organised into five sections comprising the introduction; the history and variation of human genes; natural selection and evolutionary conflicts; pathogens: resistance, virulence, variation and emergence; and, finally, non-infectious and degenerative disease. Most chapters were written by more than a single author, but there are exceptions. In section two we learn about the global spatial patterns of infectious diseases and human evolution, human genetic variation, medically relevant variation in the human genome, ecogenetic variation and its consequences for health. These were all interesting and well written. In section three we read about evolutionary conflicts in pregnancy and childhood, hormone mediated trade-offs in health and disease, the role of the major histocompatability complex in mate choice, reproductive success and disease, the evolutionary-behavioural ecologist's view on human health and disease. Again all were well written and informative. In part four we learn about antibiotic-resistant bacteria, evolution of pathogens in relation to the pressures of vaccination, the evolution and expression of virulence in infectious organisms, the evolutionary origins of diversity in human viruses, the population structure of pathogenic bacteria, and emergence of new infectious diseases. Once again a fascinating set of compilations, all very worthwhile. In the final part of the book we turn our attention to non-infectious conditions and degenerative diseases. Not surprisingly aging gets more than its fair share of attention. Metabolic disorders, the influence of lifestyle, diet on survival and chronic diseases, cancer are all considered in relevant detail.

The editors of this book set out to explain how evolutionary thought illuminates medical science. If I can have one quibble, although I admit I am biased, I would have thought that a chapter on how helminth infections have influenced and continue to influence human health and disease was relevant and, to me at least, an obvious omission. Parasitic worms are masters of immune evasion and the arms race between them and their hosts' ever increasingly sophisticated immune systems over evolutionary time make a fascinating and highly relevant story. Being infected with worms is the normal condition, and it is the absence of worm infections that has contributed to the lack of immune regulation in our hygienic developed world. The hygiene hypothesis and its various modifications propose that lack of exposure to microorganisms is responsible for the epidemic rise in allergies and autoimmune diseases in the developed world. The medical consequences of allergies are profound, and the increasing incidence of asthmatic conditions in our societies is a topic of considerable concern. Of similar concern is the rise in autoimmune diseases such as type 1 diabetes. In a book explaining the importance of evolution in understanding current medical problems, this topic should have been considered for inclusion.

Otherwise, I enjoyed reading this book, and found little else to moan about. The writing throughout is clear and interesting. Each chapter held my attention and I enjoyed the exercise. In my opinion the editors have succeeded very well indeed in their objectives and I congratulate them for a superb job. The book should be a welcomed addition to the shelves of academics and students in the biological sciences, as well as medical practitioners for whom it was primarily intended. In my case it will not be far from reach as I go through the inevitable annual cycle of revamping my lectures for the current academic year.

## Competing interests

The author declares that they have no competing interests.

